# Structure and function of mouse lens suture examined by 2-photon fluorescence microscopic imaging

**DOI:** 10.1038/s41598-026-45299-2

**Published:** 2026-03-24

**Authors:** Qinrong Zhang, Jun Zhu, Taishi Painter, Chun-Hong Xia, Na Ji, Xiaohua Gong

**Affiliations:** 1 Department of Physics, University of California, Berkeley, CA 94720 USA; 2 Department of Neuroscience, University of California, Berkeley, CA 94720 USA; 3 Herbert Wertheim School of Optometry, University of California, Berkeley, CA 94720 USA; 4 Vision Science Program, University of California, Berkeley, CA 94720 USA; 5 Helen Wills Neuroscience Institute, University of California, Berkeley, CA 94720 USA; 6 Molecular Biophysics and Integrated Bioimaging Division, Lawrence Berkeley National Laboratory, Berkeley, CA 94720 USA; 7 Department of Biomedical Engineering, College of Biomedicine, City University of Hong Kong, Kowloon, Hong Kong SAR China

**Keywords:** Biophysics, Diseases, Medical research

## Abstract

We used two-photon fluorescence microscopy (2PFM) to examine lens fiber and suture architecture, as well as permeability, in wild-type (WT) lenses and in lenses from klotho-like protein homology (KLPH) knockout (KLPH-KO) mice. KLPH is a type I membrane glycoprotein encoded by the *Lctl* gene; KLPH-KO mice develop lens suture cataracts. Lens sutures have been hypothesized to serve as pathways for transporting ions, nutrients, and other factors as part of the lens microcirculation required to maintain homeostasis and transparency. Three-dimensional (3D) in vivo imaging revealed typical “Y” and “double-Y” anterior suture geometries, along with depth-dependent variation, in WT lenses. In contrast, KLPH-KO lenses exhibited markedly heterogeneous suture morphologies and misalignment between the anterior and posterior suture planes. Quantification of pattern variability using the mean structural similarity index (SSIM) of individual z-stacks relative to the stack mean projection revealed a significant increase in pattern randomization in KLPH-KO lenses (*p* < 0.05). Both WT and KLPH-KO lenses displayed voids near sutures and enlarged vacuoles distributed throughout the lens in vivo. Notably, KLPH-KO lenses exhibited irregular, enlarged central voids containing ~ 2–5 μm amorphous structures, consistent with subcellular remnants and/or membrane-associated aggregates. In conclusion, this study provides novel morphological markers for characterizing suture cataracts and associated fiber pathology. It further demonstrates that the dye-impermeable lens suture represents a stabilized interface formed by elongated fiber ends, supporting lens integrity and maintaining the organization of lens fibers.

**Supplementary Information:**

The online version contains supplementary material available at 10.1038/s41598-026-45299-2.

## Introduction

Normal vision depends on the ocular lens, a transparent biconvex tissue with a graded refractive index located behind the iris that focuses incoming light onto the retina. The lens consists of a mass of elongated fiber cells, covered on its anterior surface by a monolayer of epithelial cells, and enclosed by a specialized basement membrane called the lens capsule. The highly ordered cellular architecture of fiber cells—and the optical properties resulting from this organization—are the primary determinants of lens transparency and refractive function^1,2^. Mammalian lenses, including those of mice and humans, continue to grow throughout life^3^. This growth is driven by the proliferation of epithelial cells near the equator and their differentiation into secondary fiber cells, which elongate and are precisely layered onto preexisting fiber “shells”^4,5^. Newly differentiating fibers extend their apical and basal ends anteriorly and posteriorly along the inner surface of the capsule toward the poles^6–8^. As elongation is completed, fiber ends detach from the capsule and meet the ends of opposing fibers at the anterior and posterior poles, forming the characteristic Y-shaped sutures along the visual axis^9^. With the successive addition of fiber layers, each cohort of secondary fibers contributes a new growth shell. Concurrently, interior fiber cells undergo maturation, involving the elimination of nuclei and other organelles, producing an organelle-free zone that minimizes light scattering and supports high optical quality^2,10^.

The development, architecture, and function of lens sutures are tightly linked to the lens’s ability to maintain transparency, precisely focus light, and adjust optical power throughout life^11–13^. Sutures form where opposing fiber-cell tips meet at the poles^9^, and they are reinforced by specialized junctional networks and cytoskeletal microfilaments^14^. By helping to preserve ordered fiber packing, sutures contribute to lens shape and mechanical integrity during accommodation and can influence overall optical performance^12,15^. Sutures have also been proposed to participate in lens microcirculation by shaping extracellular spaces that support fluid movement and nutrient delivery^13,16^. Consistent with these roles, disrupted fiber-cell organization and abnormal suture formation are frequently associated with increased light scattering and cataractogenesis^17–19^. However, most cellular and molecular features of suture pathology have been characterized in dissected or fixed lenses ex vivo, which can obscure native 3D organization and introduce preparation artifacts. High-resolution imaging approaches that enable direct visualization of living lenses are therefore essential for defining in vivo cellular and subcellular pathological changes in their native state.

Optical microscopy, owing to its subcellular resolution and non-invasiveness, has become a powerful tool for studying living organisms. Among conventional imaging modalities, 2-photon fluorescence microscopy (2PFM) possesses unique advantages for studying the mouse retina^20,21^ and the lens^22^. The nonlinear absorption process provides intrinsic optical sectioning capabilities to 2PFM, allowing depth-resolved 3D imaging of the mouse eye lens. Moreover, utilizing near-infrared (NIR) excitation, which scatters less, 2PFM is well-suited for studying normal and pathological lenses. One challenge in in vivo 2PFM mouse eye imaging is optical aberrations introduced by the imperfect mouse eye, resulting in degraded resolution and contrast. By incorporating adaptive optics (AO), we recently demonstrated the first 2PFM imaging of the mouse eye lens in vivo and discovered previously unreported lens features^22^. However, this AO-2PFM system had a limited imaging field of view (FOV) and imaging speed, which did not fully capture the organization of lens fiber cells nor provide significant insights into the diseased mouse model.

Using a 2PFM platform equipped with resonant scanners to enable higher-speed acquisition over a larger FOV, we examined the structure and function of lens sutures between WT and KLPH-KO lenses. Klotho-like protein homology (KLPH), encoded by *Lctl* (also known as lactase-like or γ-Klotho), is a type I membrane glycoprotein and a member of the Klotho family. Previous studies suggest that KLPH is essential for normal lens suture formation and influences lens microcirculation and transport^23^. Mutations in the klotho gene in mice cause a premature aging syndrome. In the lens, KLPH transcripts are highly expressed in the epithelium and elongating fibers^23,24^. KLPH-KO lenses develop age-related cortical cataracts and display loose or open double-Y (double opposing “Y”)- or X-shaped lens sutures, compared to normal Y-shaped sutures^23^. Thus, these features make KLPH-KO mice a valuable model for testing the hypothesis that lens sutures influence lens microcirculation and transport^16^.

The results of these in vivo imaging experiments in live mice reveal various lens suture pathological features that were underappreciated with previous imaging methods, including reduced light transmission efficiency, disorganized suture lines, larger and multiple voids at the suture junction, side voids in addition to the central voids, and disorganized fiber cells undergoing degradation. The imaging data reveal that the central void area, which was observed but whose causes were unknown in our previous study^22^, is composed mostly of healthy (i.e., with intact membrane integrity) cells, along with degrading cells and extracellular space. To our knowledge, this work represents the first in vivo 2PFM investigation of fine pathological features in lens sutures and provides new morphological markers for evaluating lens optical pathology and suture cataracts. We further examined whether the diffusion pathway of FITC-Dextran is present along the lens anterior suture region using ex vivo imaging of FITC-Dextran-incubated lenses.

## Results

### 2PFM imaging of lens fiber cell organization and alterations in KLPH-KO lenses

Lens sutures in mice typically exhibit characteristic “Y” or “double-Y” patterns^25,26^. Three-dimensional imaging of these suture structures has been challenging due to the limited optical sectioning capability of conventional widefield microscopy. Although confocal fluorescence microscopy provides optical sectioning capability, its application has been restricted to dissected, thin lens sections due to limited penetration, except when using longer wavelengths^27^. Since a previous study reported that KLPH-KO lenses exhibited a loose suture defect based on in vitro images^22^, we aimed to use a 2PFM system with resonant scanners to achieve higher imaging speed over a larger field of view and to examine the 3D suture structures in both normal and loose-suture lenses. In addition, we sought to evaluate whether this in vivo lens imaging technology could be used to test the long-standing hypothesis that the lens suture provides an effective diffusion pathway for maintaining lens homeostasis in the lens core^16,28^.

**Fig. 1:**
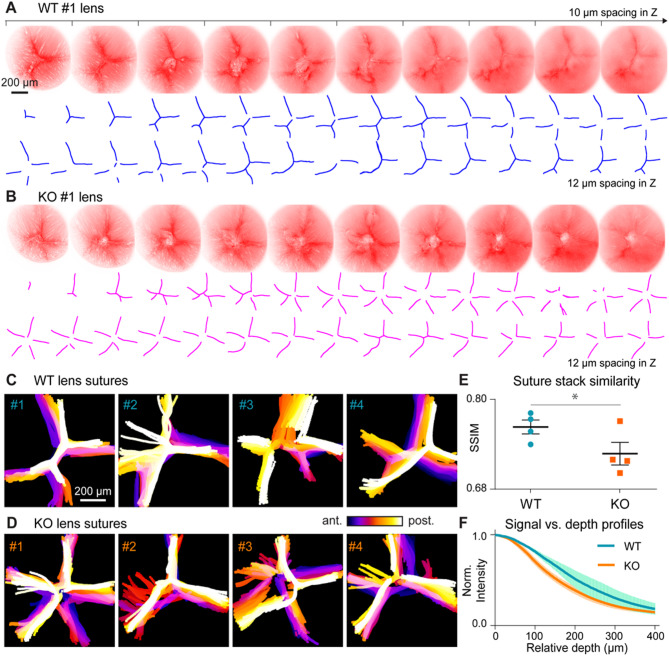
Comparison of suture organization in wild-type (WT) and KLPH-KO lenses. (**A**,**B**) Representative 2PFM images of sutures (top) and manually extracted suture lines (bottom) at 10–12 μm depth intervals from a WT (**A**) and a KLPH-KO (**B**) lens, respectively. Imaging field of view: 796.36 μm × 796.36 μm. (**C**,**D**) Depth-encoded projections of manually outlined suture patterns from WT and KLPH-KO lenses, respectively. (**E**) Quantification of suture pattern variations, expressed as the mean structural similarity index (SSIM) of images at each depth relative to the mean projection. Error bars represent ± standard deviation, and the p value was determined using an unpaired, one-sided Student’s t-test. (**F**) Normalized signal levels from WT and KLPH-KO lenses as a function of depth. Signal levels were normalized to the illumination power and applied PMT gain. Shaded areas represent the standard deviation of the measurements. Mice were 7–8 months old at the time of in vivo imaging; see Table S1 for details.

By utilizing 2PFM, we performed in vivo imaging of age-matched mouse lenses over large volumes (approximately 800 × 800 × 800 µm^3^), revealing that suture patterns vary at different depths along the optical axis in both WT and KLPH-KO mice, as also observed in their depth-encoded projections. Consistent with previously published studies^25,26^, WT lens sutures exhibited either “Y” or “double-Y” patterns at different depths, roughly corresponding to their “double-Y” suture envelopes (Fig. 1A, C, Figs. S1, S3, and Movie 1). In contrast, KLPH-KO mice displayed diverse suture patterns, including “Y”, “double-Y”, and “star”, with variations appearing more randomly at different depths and misaligned suture planes (Fig. 1B, D, Figs. S2, S4). The variability in suture patterns was quantified using the mean structural similarity index (SSIM) of image stacks relative to the stack’s mean projection, revealing a significant increase (*p* < 0.05) in pattern randomization for KLPH-KO lenses (Fig. 1E).

This increased complexity and randomness in the suture structures of KLPH-KO mice may reduce light transmission through the lens. Indeed, the fluorescence signal from KLPH-KO lenses decreased more rapidly than that from WT lenses, suggesting compromised lens transparency and a potential impact on vision quality (Fig. 1F).

### Characterization of voids and vacuoles along the lens suture in FITC-Dextran-incubated lenses in vitro

Our previous work^22^, applying 2PFM for in vivo imaging of mouse lenses, identified large voids and enlarged vacuoles in the anterior region of KLPH-KO mouse lenses. High-resolution 2PFM further confirmed the presence of these voids and vacuoles; however, whether they are intracellular or extracellular structures remains undetermined.

**Fig. 2:**
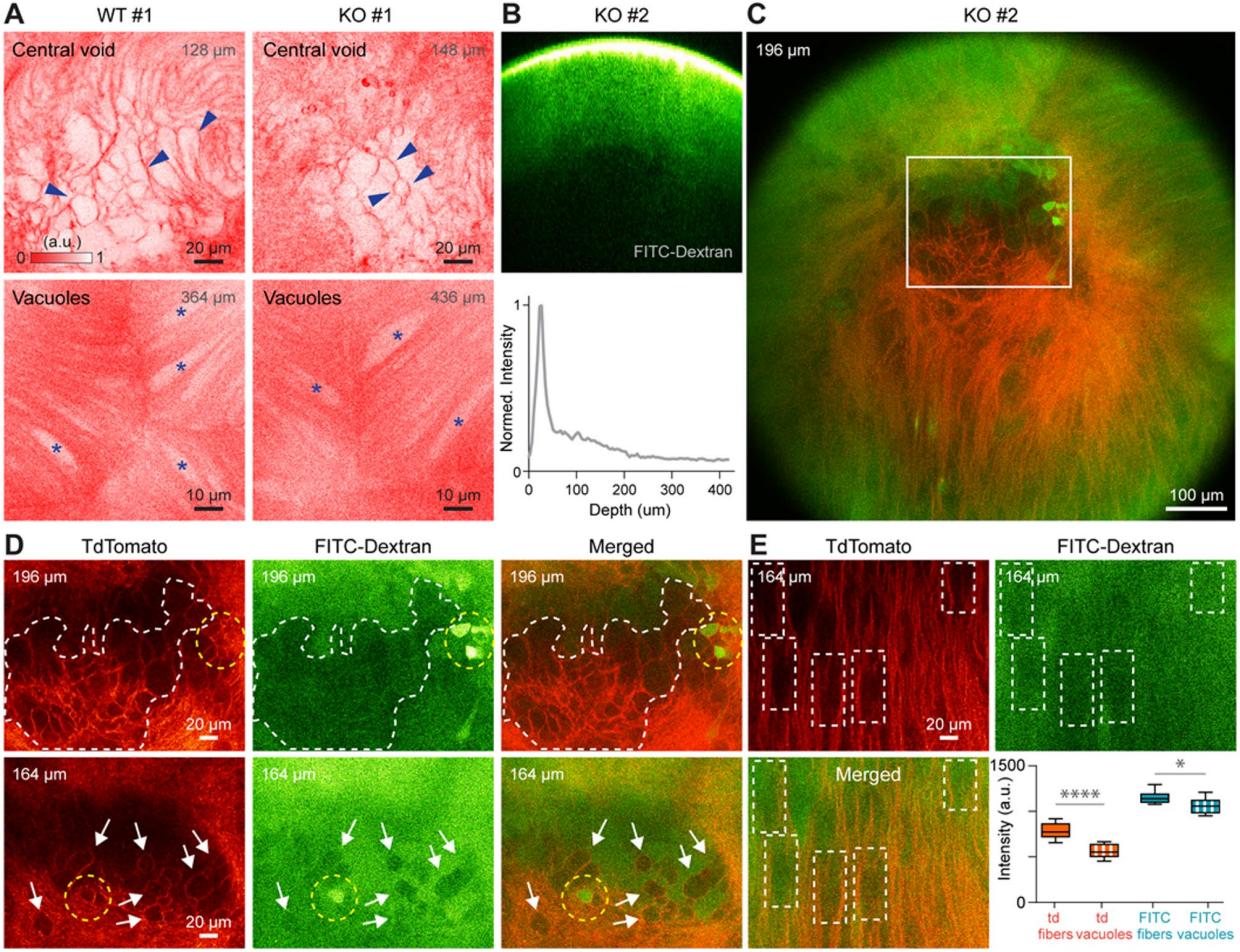
2PFM imaging of central voids and vacuoles in the mouse lens. (**A**) In vivo single-plane 2PFM images of the central region at the conjunction of suture lines, acquired from a WT (left) and a KLPH-KO (right) mouse lens. Central voids (blue arrowheads) and vacuoles (asterisk) are highlighted. (**B**) Top: cross-sectional view of a FITC-Dextran-incubated KLPH-KO lens. Bottom: green fluorescence signal measured by 2PFM. (**C**) Merged two-color 2PFM image of a tdTomato-labeled KLPH-KO lens incubated with FITC-Dextran. (**D**) Single-plane 2PFM images of the central region of a KLPH-KO lens at the conjunction of suture lines in the red (left) and green (middle) channels, as well as the merged image (right), acquired at depths of 196 μm (upper) and 164 μm (lower), respectively. (**E**) Single-plane 2PFM images of vacuoles in a KLPH-KO lens, acquired in red and green channels, as well as the merged image. Bottom right: quantitative comparisons of fiber and vacuole signals, based on the mean intensity calculated from nine fiber and nine vacuole regions measured in the red and green channels, respectively. All images were adjusted for improved visualization. Mice were 7–8 months old at the time of in vivo imaging (**A**) and 13 months old at the time of ex vivo imaging (**B**–**E**); see Table S1 for details.

By imaging WT and KO mice, we observed that both lenses exhibit voids where sutures meet, as well as enlarged vacuoles throughout the lens volume in vivo (Fig. 2A). To investigate the physiological nature of these structures at the lens suture and their potential involvement in intralenticular diffusion pathways, we incubated freshly enucleated WT and KO lenses in FITC-Dextran solution overnight before 2PFM imaging. The results showed that the green dye successfully penetrated the lens, reaching a depth of over 300 μm (Fig. 2B). Since FITC-Dextran (MW 2 M/10K) is impermeable to the lens cell plasma membrane, which is labeled with tdTomato in both WT and KO mice, the diffused FITC-Dextran signal detected by two-color 2PFM imaging was mainly localized to extracellular spaces, with a few exceptions in which some lens epithelial or fiber cells appeared FITC-Dextran-positive, likely due to cell damage. We speculated that such cell damage might be associated with the long in vitro incubation of fresh lenses with dye. However, it remains unclear how FITC-Dextran entered those cells (Fig. 2C).

At a depth of 196 μm from the lens surface, we observed that the central void largely corresponds to an area with a globally decreased green signal (Fig. 2D, upper middle, white dashed shape). We speculated that “voids” in this region, defined by tdTomato-labeled plasma membranes (Fig. 2D, upper left, white dashed shape) and minimal FITC-Dextran labeling, likely represent intracellular regions of tightly tethered fiber cell bundles with impermeable extracellular spaces. Notably, a few cells at the same depth near this region were labeled with intense FITC-Dextran green signals (Fig. 2D, upper row, yellow circles), likely resulting from limited cell damage during in vitro incubation. Similar observations were made at another depth (164 μm), where some cells had lower signals (Fig. 2D, lower row, white arrows) and one cell was labeled with FITC-Dextran (Fig. 2D, lower row, yellow circle). These findings suggest that the central region, or central voids, formed at the conjunction of the suture lines, mainly comprises tightly apposed, irregularly shaped ends of lens fiber cells, with only very few fiber cells (likely damaged or unhealthy) showing positive FITC-Dextran dyes (Fig. 2 and Fig. S5).

Two-color 2PFM imaging also revealed that the enlarged “vacuoles” contained reduced green signals compared to adjacent lens fibers (Fig. 2E, five dashed white boxes). The mean fluorescence intensity calculated from nine fiber and nine vacuole regions confirmed that these “vacuoles” exhibited reduced green FITC-Dextran and tdTomato signals (Fig. 2E, lower right). Since normal mouse lenses are composed of elongated fibers with minimal extracellular spaces^29,30^, lens fibers are unlikely to form these “vacuoles”. Therefore, we speculate that these “vacuoles” likely result from lens fiber cell regions containing less membrane-tagged tdTomato, in which tightly opposed fiber ends reduce extracellular permeability to FITC-Dextran.

### Pathological features in KLPH-KO lenses

**Fig. 3:**
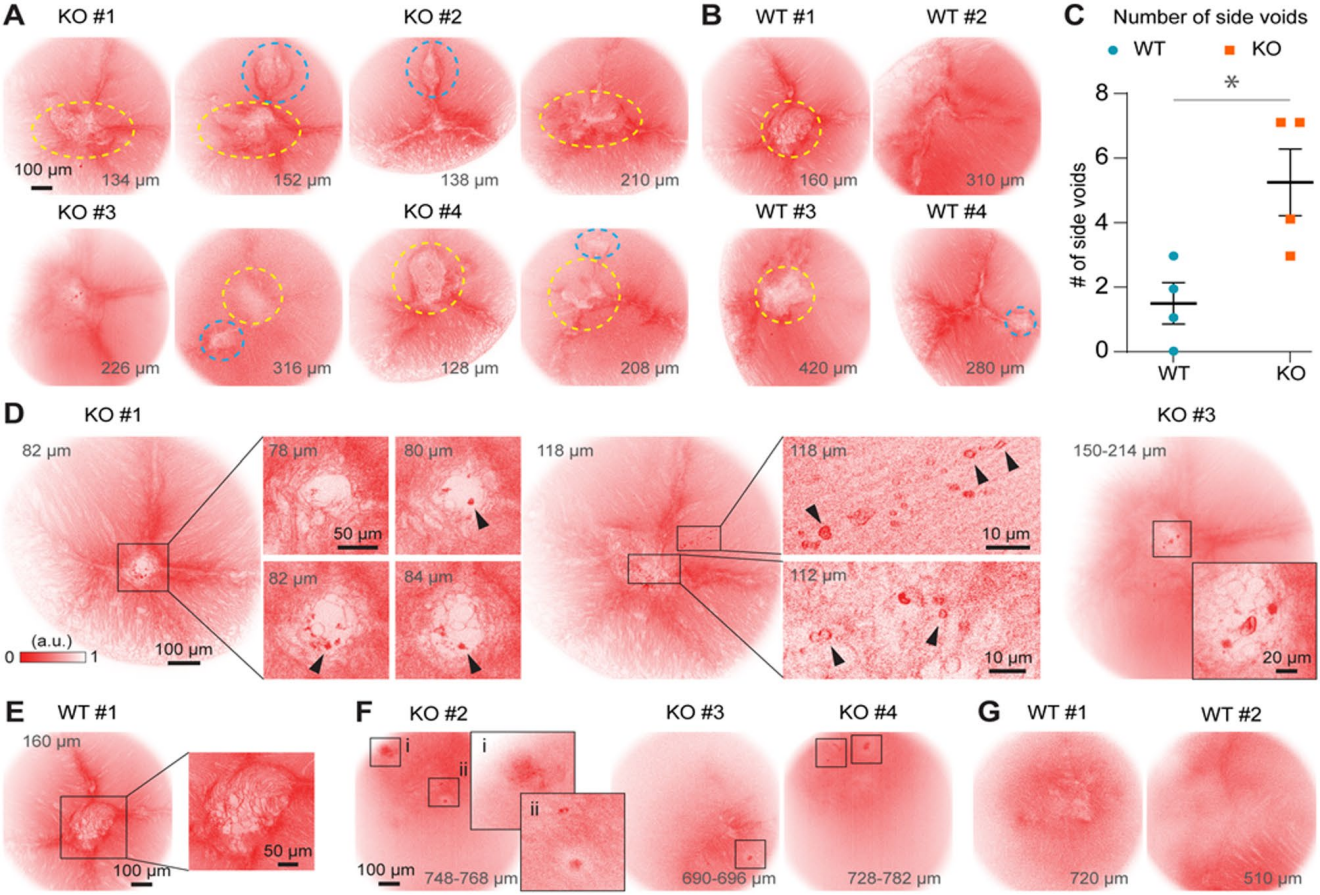
The 2PFM imaging of KLPH-KO and WT lenses. (**A**) Four KLPH-KO lenses showing central and side voids formed at the central conjunction of suture lines (yellow circles) and between suture lines (cyan circles), respectively. Fewer side voids are observed in four WT lenses (**B**), and this difference was confirmed by an unpaired one-sided Student’s t-test (**C**). (**D**,**E**) Representative images from two KLPH-KO lenses showing degraded fiber cells (black arrowheads), a feature not observed in WT lenses. (**F**,**G**) Representative images at deeper depth showing degraded fiber cells in KLPH-KO lenses but not in WT lenses. Mice were 7–8 months old at the time of in vivo imaging; see Table S1 for details.

Lens sutures are formed by the contacts of the ends of elongated fiber cells. Disrupted contact stability and/or altered morphological or biophysical properties at the ends of these cells are likely to cause suture cataract formation in KLPH-KO lenses. We therefore focused on in vivo imaging of this suture region to identify pathological features in KLPH-KO lenses compared with WT controls (Fig. 3).

Comparing four KLPH-KO lenses (Fig. 3 and Fig. S2) with four WT lenses (Fig. 3, Figs. S1 and S6), we observed that KLPH-KO lenses contained at least one additional void within the imaging volume (Fig. 3A, cyan dashed circles), in addition to the central void formed at the conjunction of suture lines (Fig. 3A, yellow dashed circles), whereas WT lenses exhibited fewer additional voids (Fig. 3B, C). These additional voids may further impair light transmission and could progress into regions containing pathological fiber cells. We also observed that KLPH-KO lenses exhibited larger central voids with irregular shapes. Within these voids, a striking pathological feature was identified: the formation of 2–5 μm-diameter ring-like or amorphous structures (Fig. 3D, insets, black arrowheads). Given the membrane-tethered tdTomato proteins, we speculate that these structures are membrane protein remnants or aggregates from pathological fiber cells. Moreover, these features were observed both within and outside the central void region (Fig. 3D, insets at 118 μm depth, mouse KO #1). In contrast, no such structures were observed in the four WT lenses imaged (Fig. 3E, Figs. S1 and S6). Furthermore, in deeper regions of KLPH-KO lenses, small areas of concentrated fluorescence were observed (Fig. 3F), a feature not seen in the WT lenses (Fig. 3G and Fig. S6).

## Conclusion and discussion

The intrinsic optical sectioning of two-photon fluorescence microscopy (2PFM) enabled depth-resolved visualization of lens anterior sutures and fiber-cell organization in vivo, revealing structural features that are difficult to capture with conventional widefield imaging. By quantitatively analyzing 2PFM image stacks, we found that KLPH-KO lenses exhibit substantially greater depth-dependent variability in suture geometry, including increased diversity of patterns, higher branching complexity, and poorer registration of suture planes across depth, compared with WT lenses. These changes were accompanied by pathological remnants at fiber ends within or adjacent to suture regions, suggesting local failure of fiber-end integration. Because suture discontinuities and irregular branch geometry introduce refractive-index discontinuities and microstructural interfaces along the optical axis, the increased complexity and irregularity observed in KLPH-KO sutures are likely to reduce light transmission and promote light scattering, thereby contributing to lens opacity. Collectively, these data support the central conclusion that KLPH is required for normal suture formation and that disrupted sutures represent a plausible structural mechanism contributing to cataract formation in KLPH-KO lenses.

Lens suture formation relies on the continual addition of concentric layers of secondary fibers throughout life. Newly elongated fibers extend from the equator toward the poles, and their tips stack onto earlier generations, generating Y-shaped branch patterns that can increase in complexity as the lens grows^9^. The molecular and cellular mechanisms that control lens suture formation are far from fully understood. Previous studies show that KLPH is required for expression of Clic5, which can associate with cytoskeleton ERM (ezrin, radixin, and moesin)-actin complexes^31,32^, which might play roles in fiber cell morphogenesis at the lens suture. Since both KLPH and Clic5 proteins are detected in lens epithelial cells^23^, a KLPH-Clic5-cytoskeleton axis is hypothesized to control elongating fiber cell morphogenesis underneath the epithelium. KLPH might also regulate the MAPK/ERK signaling pathway in human lens epithelial cells^24^. However, neither the location nor the distribution of KLPH and Clic5 proteins is known in lens fiber cells. How KLPH deficiency leads to lens suture defects remains to be investigated. Eph-ephrin signaling is essential for suture patterning, since both EphA2 knockout and ephrin-A5 knockout lenses display misalignment of lens sutures^18,25^, as well as reduced suture gaps but increased lens resilience under mechanical compression^26^. However, like KLPH-KO lenses, the molecular mechanisms by which deficiency of either EphA2 or ephrin-A5 alters lens suture alignment remain unknown.

To test whether suture abnormalities correspond to an “open” extracellular conduit, we performed ex vivo 2PFM imaging following incubation of intact lenses in FITC-Dextran. Across both WT and KLPH-KO lenses, high-molecular-weight dextran did not reveal an obvious diffusion pathway along the suture lines. Instead, dye penetration was most consistent with diffusion through extracellular spaces of elongating or elongated fibers, reaching > 300 μm into the lens (Fig. 2B). Importantly, we did not observe an open suture in KLPH-KO lenses, either in vivo or following ex vivo incubation, in contrast to a previous report that relied on fixed-lens imaging^23^. Thus, our results argue against the model in which normal or KLPH-KO lenses form a persistent, macroscopically open suture that serves as a major extracellular diffusion pathway for solutes entering the lens.

Higher-resolution 2PFM images further revealed that the “central void” regions at suture junctions are structurally heterogeneous, comprising tightly apposed fiber cells with irregular ends and narrower extracellular spaces, which further support the conclusion that lens sutures are impermeable to dextran dyes. In KLPH-KO lenses, tdTomato-positive remnants and aggregates were enriched along suture regions, consistent with fiber-end pathology as a hallmark feature of the KO phenotype. These structures provide new, image-based morphological markers for cataract-associated pathology in vivo and may be particularly useful as outcome measures for future mechanistic or interventional studies.

Beyond suture-localized defects, we also observed enlarged vacuole-like structures at multiple depths in live KLPH-KO lenses. These features often appeared as discrete regions associated with bundles of several fibers (on the order of 4–5 fibers, based on apparent diameters) that showed reduced tdTomato labeling and altered local extracellular spacing. While the present imaging data do not yet define their cellular origin, these structures are unlikely to be explained solely by uniform fiber-cell hypertrophy in selective regions or by simple plasma-membrane fusion events previously described for certain lens fiber compartments^33^. We cannot rule out potential effects of membrane-targeted tdTomato in lens fibers, nor can we exclude a direct contribution of tdTomato to the vacuole-like structures observed in wild-type, tdTomato-expressing clear lenses in vivo. Together with the presence of larger and more irregular central voids and multiple void-like regions containing membrane-associated remnants, these observations suggest that KLPH may contribute to fiber-end integration at the lens suture. This work further suggests that lens suture architecture and alignment influence the broader structural integrity and stability of elongated lens fiber cells, consistent with previous studies of EphA2KO and ephrin-A5KO lenses^26^. The molecular mechanisms by which KLPH supports suture pattern formation and fiber stability remain unresolved and will require further investigation, including molecular analyses of candidate adhesion and cytoskeletal pathways at fiber ends^14^.

In summary, this study demonstrates the power of 2PFM for revealing the three-dimensional organization of lens fiber cells and for identifying in vivo pathological features associated with cataract formation. Our findings support the concept that lens sutures form a stabilized end-to-end interface, maintaining lens architecture as a mechanically integrated syncytium, which may be important for preserving optical quality during accommodation-related deformation. The structural abnormalities observed in KLPH-KO lenses–disorganized, depth-variable sutures and fiber-end remnants–provide a mechanistic link between disrupted cellular organization and reduced transparency, and they establish quantitative, image-based biomarkers that can be used in future studies aimed at preventing or reversing cataract-associated pathology in vivo.

## Methods and materials

### Mouse models

All animal experiments, including mouse care and breeding, were conducted in accordance with the Animal Welfare Regulations, the National Institutes of Health (NIH) guidelines and regulations for using animals in research, and the ARVO Statement for the Use of Animals in Ophthalmic and Vision Research. The animal experimental protocols, including animal care, monitoring, and breeding, were approved by the Institutional Animal Care and Use Committee (IACUC) at the University of California, Berkeley. This study is reported in accordance with ARRIVE guidelines for animal research. Mice were housed with free access to food and water under a 12:12 h light: dark cycle. Euthanasia was performed by CO_2_ inhalation followed by cervical dislocation.

The membrane-targeted tdTomato-expressing mice (007676, ROSA^mT/mG^) were obtained from the Jackson Laboratory and are congenic on the C57BL/6J background. Klotho-related protein KLPH (lctl) knockout (KO) mice were kindly provided by Dr. Melinda Duncan at the University of Delaware. The KLPH-KO mice were originally generated by Dr. Graeme Wistow at the National Eye Institute. In this study, membrane-targeted tdTomato-expressing mice were used as the WT controls^34^, and backcrossed tdTomato-expressing KLPH-KO mice served as the diseased group^23^. All mice were maintained on the C57BL/6J genetic background. No noticeable differences in body size or weight were observed among the mice. KLPH-KO mice grew normally, comparable to their WT littermates. All in vivo imaging experiments were performed on mice at 7 or 8 months of age, with body weights of approximately 25–30 g. Genotype, sex, and age at imaging are summarized in Supplementary Table S1.

### 2-photon fluorescence microscope (2PFM)

For in vivo mouse lens imaging, a commercial 2PFM system (Thorlabs Bergamo^®^ II multiphoton) was used. A femtosecond Ti: Sapphire laser (Coherent, Chameleon Ultra II) was used as the excitation source for all experiments. The laser was tuned to 1000 nm for in vivo imaging of tdTomato-expressing mouse lenses, and to 920 nm for ex vivo imaging of FITC-Dextran-incubated tdTomato-expressing lenses. A 25× water-dipping objective lens (Olympus, 1.05 NA, 2 mm WD) was used to focus the excitation light into the lens and collect the emitted fluorescence for imaging. Data acquisition and hardware were controlled using the ThorImage software. Imaging experiment settings are summarized in Supplementary Table S1.

### In vivo mouse lens imaging

In vivo imaging experiments were performed on mice under light isoflurane anesthesia (0.5-1% by volume in O_2_). Prior to imaging, each mouse was stabilized on a bite bar mounted on a custom-made stage with a dual-axis goniometer (Thorlabs, GNL 18) and aligned so that the pupil faced up. The pupil was dilated with 2.5% phenylephrine hydrochloride (one drop, Paragon BioTeck, Inc.) and 1% tropicamide (one drop, Akorn, Inc.). To prevent corneal drying and clouding, eye gel (Genteal) was applied between the eye and a cover glass (Fisherbrand^®^, No. 1.5, 0.16–0.19 mm thick). The cover glass was mounted on a holder to minimize the effect of eye motion and carefully aligned to be perpendicular to the excitation light. During imaging, the mouse’s body temperature was maintained using a hand warmer. The correction collar of the objective lens was adjusted to minimize spherical aberration induced by the cover glass.

### Ex vivo mouse lens imaging

Eyes were retrieved and dissected in culture medium composed of M199 (Invitrogen, Carlsbad, CA) supplemented with HEPES (Invitrogen, Carlsbad, CA) at 37 °C to extract the lenses. Dissected lenses were then incubated in the culture medium containing either 2,000,000 MW FITC-Dextran (Sigma-Aldrich, St. Louis, MO) or 10,000 MW FITC-Dextran (Molecular Probes, Eugene, OR) at various concentrations. Lenses were incubated at 37 °C in 5% CO_2_ and 95% humidity for 20–24 h (Supplementary Table S1). After incubation, lenses were washed three times with culture medium before imaging. During imaging, lenses were placed in wells containing M199 culture medium as the immersion medium.

### Image processing and analysis

All image processing, visualization, and analysis were performed using ImageJ (NIH, Bethesda, MD, USA)^35^ and MATLAB (Mathworks, Natick, MA, USA). Suture lines were manually traced at different depths using ImageJ. For Fig. 1F, the two-photon fluorescence intensity measured at each imaging depth was first normalized to the excitation laser power delivered to the sample and to the photomultiplier tube (PMT) gain. This correction compensates for depth-dependent differences in excitation intensity and detector amplification, ensuring that the resulting values primarily reflect depth-dependent attenuation rather than variations in acquisition settings. The corrected signals were then normalized to the surface intensity, allowing comparison of relative fluorescence decay across samples as a function of imaging depth.

## Supplementary Information

Below is the link to the electronic supplementary material.

Supplementary Material 1

Supplementary Material 2

Supplementary Material 3

## Data Availability

All data generated or analyzed in this study are included in the manuscript and supplementary materials. The datasets used and/or analyzed during the current study are available at Figshare (10.6084/m9.figshare.30551129) or from the corresponding author upon reasonable request.
