# Supplementary material for: Structure and function of mouse lens suture examined by 2-photon fluorescence microscopic imaging
Source: Sci Rep. 2026 Mar 24;16:14788. doi: 10.1038/s41598-026-45299-2 (PMC13168428; doi:10.1038/s41598-026-45299-2)
Supplement: Supplementary file 3 — Supplementary Material 3 [file 41598_2026_45299_MOESM3_ESM.docx]

Supplementary Materials for

**Structure and Function of Lens Suture Examined by 2-photon Fluorescence Microscopic Imaging**

**Qinrong Zhang^1,7,#^, Jun Zhu^2,#^, Taishi Painter^3,4^_,_ Chun-Hong Xia^3,4^, Na Ji^1,2,5,6^, Xiaohua Gong^3,4,^** ^*^

^1^Department of Physics, University of California, Berkeley, CA 94720, USA

^2^Department of Neuroscience, University of California, Berkeley, CA 94720, USA

^3^School of Optometry, University of California, Berkeley, CA 94720, USA

^4^Vision Science Program, University of California, Berkeley, CA 94720, USA

^5^Helen Wills Neuroscience Institute, University of California, Berkeley, CA 94720, USA

^6^Molecular Biophysics and Integrated Bioimaging Division, Lawrence Berkeley National Laboratory, Berkeley, CA 94720, USA

^7^Present address: Department of Biomedical Engineering, College of Biomedicine, City University of Hong Kong, Hong Kong SAR

^#^These authors contributed equally

^*^*Corresponding author:* [*xgong@berkeley.edu*](mailto:xgong@berkeley.edu)

**This file includes:**

Figure S1. 2PFM lens images of the wild-type (WT) mice acquired at different depths.

Figure S2. 2PFM lens images of the KLPH-KO mice acquired at different depths.

Figure S3. Suture lines of the wild-type (WT) mice at different depths.

Figure S4. Suture lines of the KLPH-KO mice at different depths.

Figure S5. 2PFM imaging of central voids in wild-type WT #5 and WT #6 mouse lenses.

Figure S6. 2PFM imaging of four wild-type (WT) lenses at different depths with void structure highlighted.


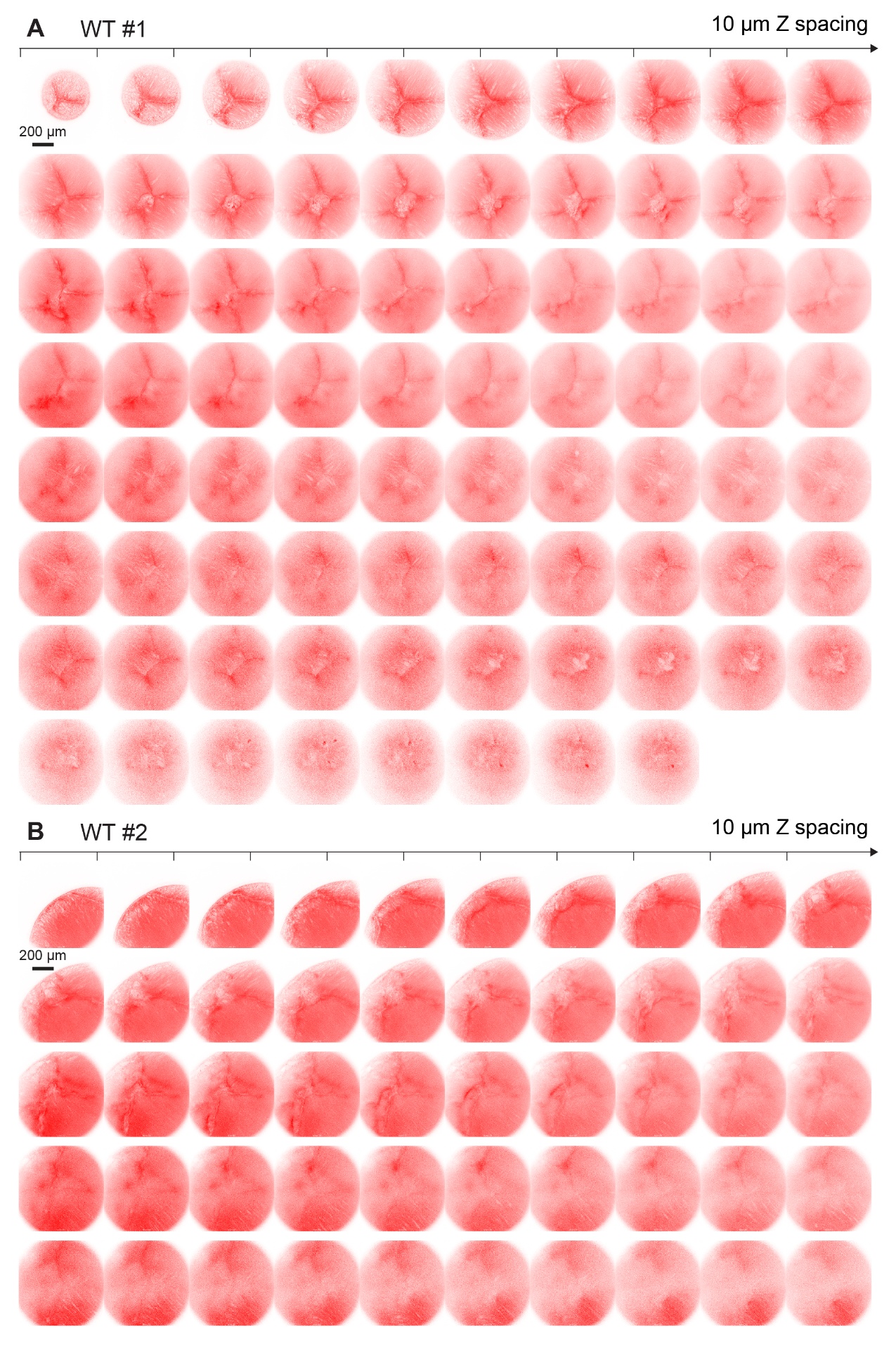


**Figure S1. 2PFM lens images of the wild-type (WT) mice (7–8 months old) acquired at different depths.**


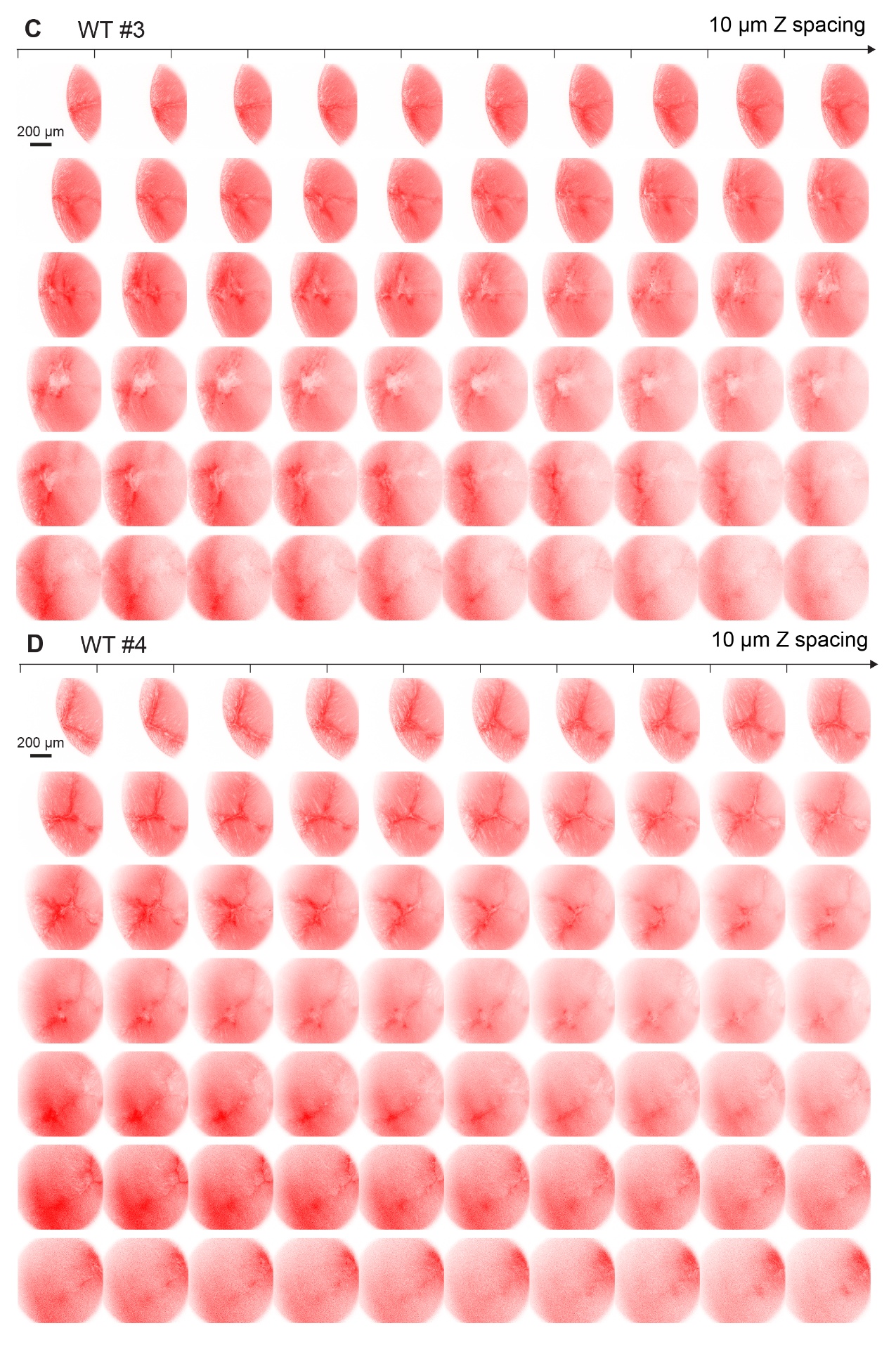


**Figure S1. 2PFM lens images of the wild-type (WT) mice (7–8 months old) acquired at different depths.**

**Figure S2. 2PFM lens images of the KLPH-KO mice (7–8 months old) acquired at different depths.**


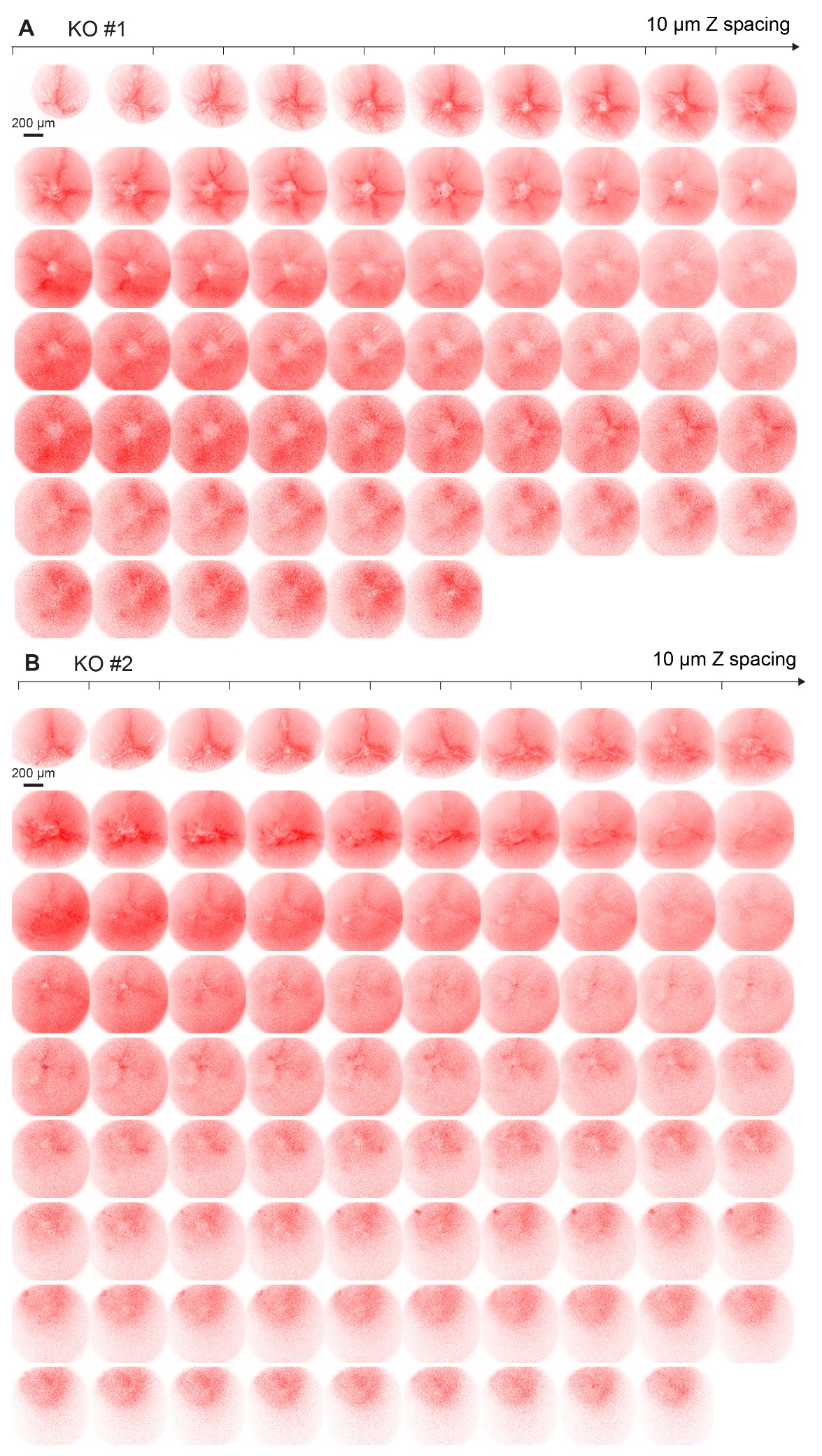


**Figure S2. 2PFM lens images of the KLPH-KO mice (7-8 months old) acquired at different depths.**


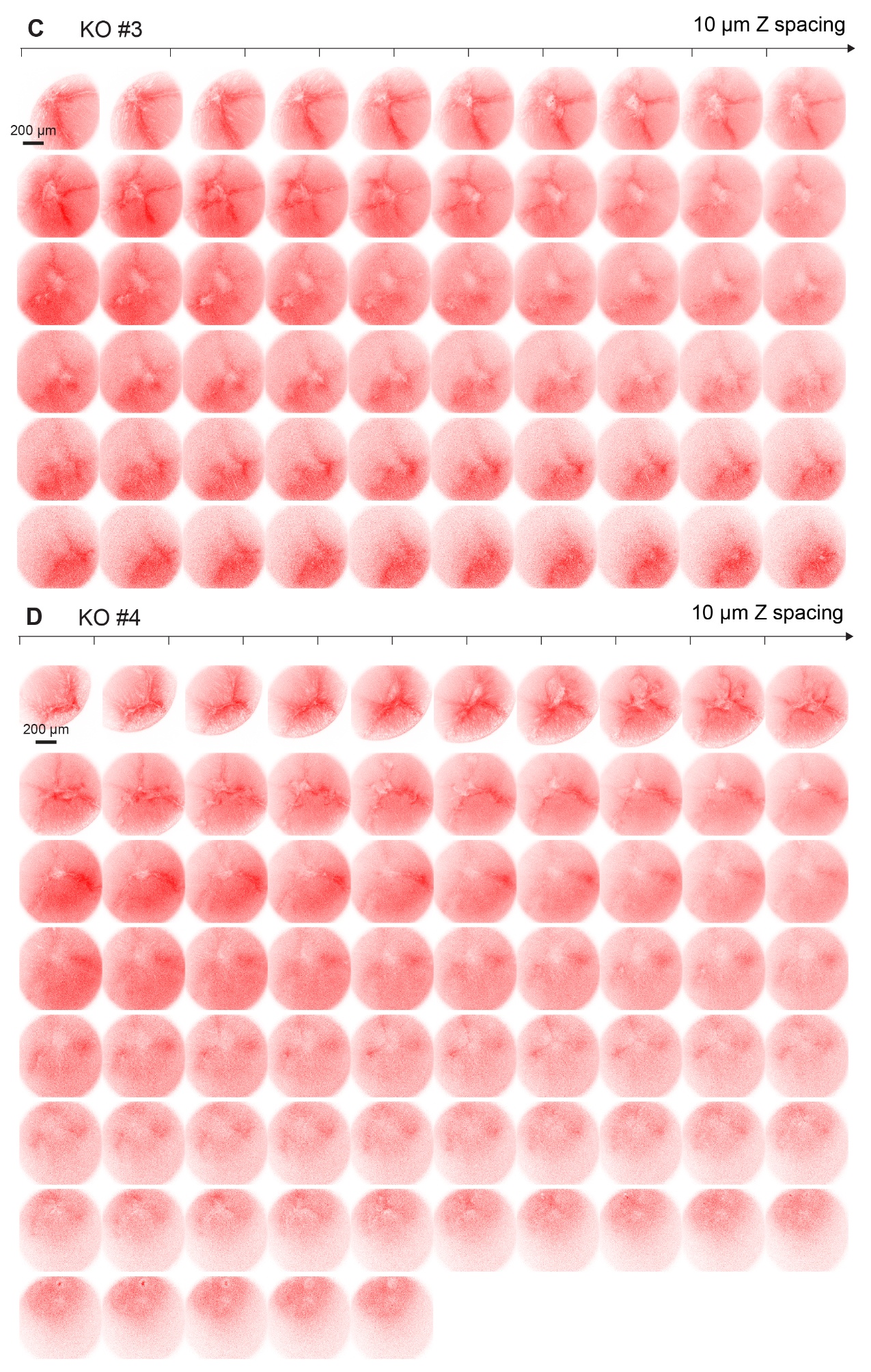


**Figure S3. Suture lines of the wild-type (WT) mice (7–8 months old) at different depths. (A-C)** Left: Depth-encoded projections of hand-drawn suture stacks from three other WT mice. Right: Suture lines at various depth at 10 µm spacing in Z. Scale bar represents 200 µm and applies to all the images.


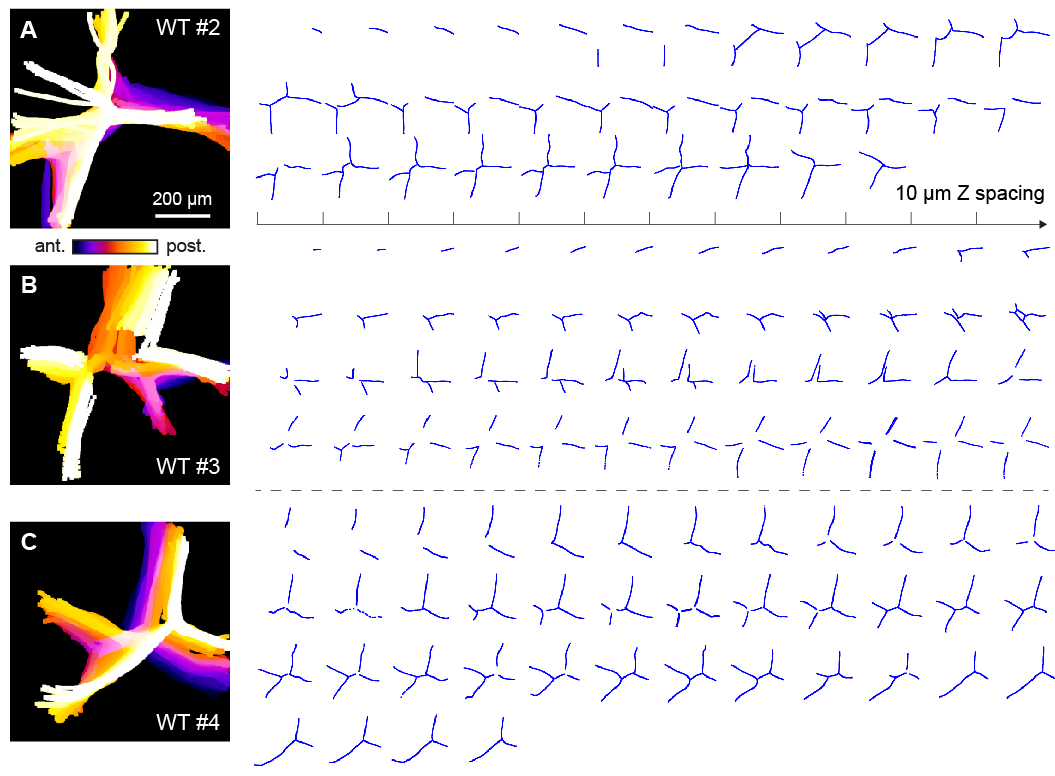


**Figure S4. Suture lines of the KLPH-KO mice (7–8 months old) at different depths. (A-C)** Left: Depth-encoded projections of hand-drawn suture stacks from three other KO mice. Right: Suture lines at various depth at 10 µm spacing in Z. Scalebar represents 200 µm and applies to all the images.


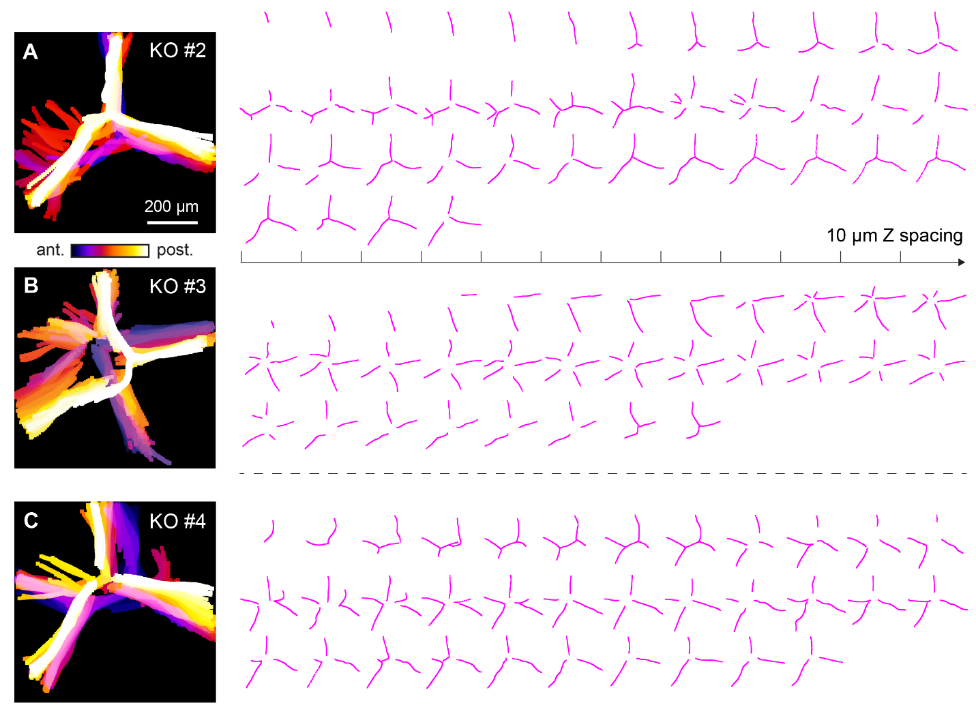


**Figure S5. 2PFM imaging of central voids in WT #5 and WT #6 mouse lenses.** (A-B) Merged 2-color 2PFM images of WT lenses incubated with FITC-Dextran. (C-F) Single-plane 2PFM images of the lens suture conjunction region (white boxes in A and B) at different depths. White arrows denote FITC signal in the green channel but not in the red channel, indicating dye penetration ability but without entering the suture conjunction area. Left: TdTomato imaged in the red channel. Middle: FITC-Dextran imaged in the green channel. Right: Merged images. Scale bars: 100 µm. Mice were 8 months old (A) and 3 months old (B) at the time of *ex vivo* imaging; see Table S1 for details.


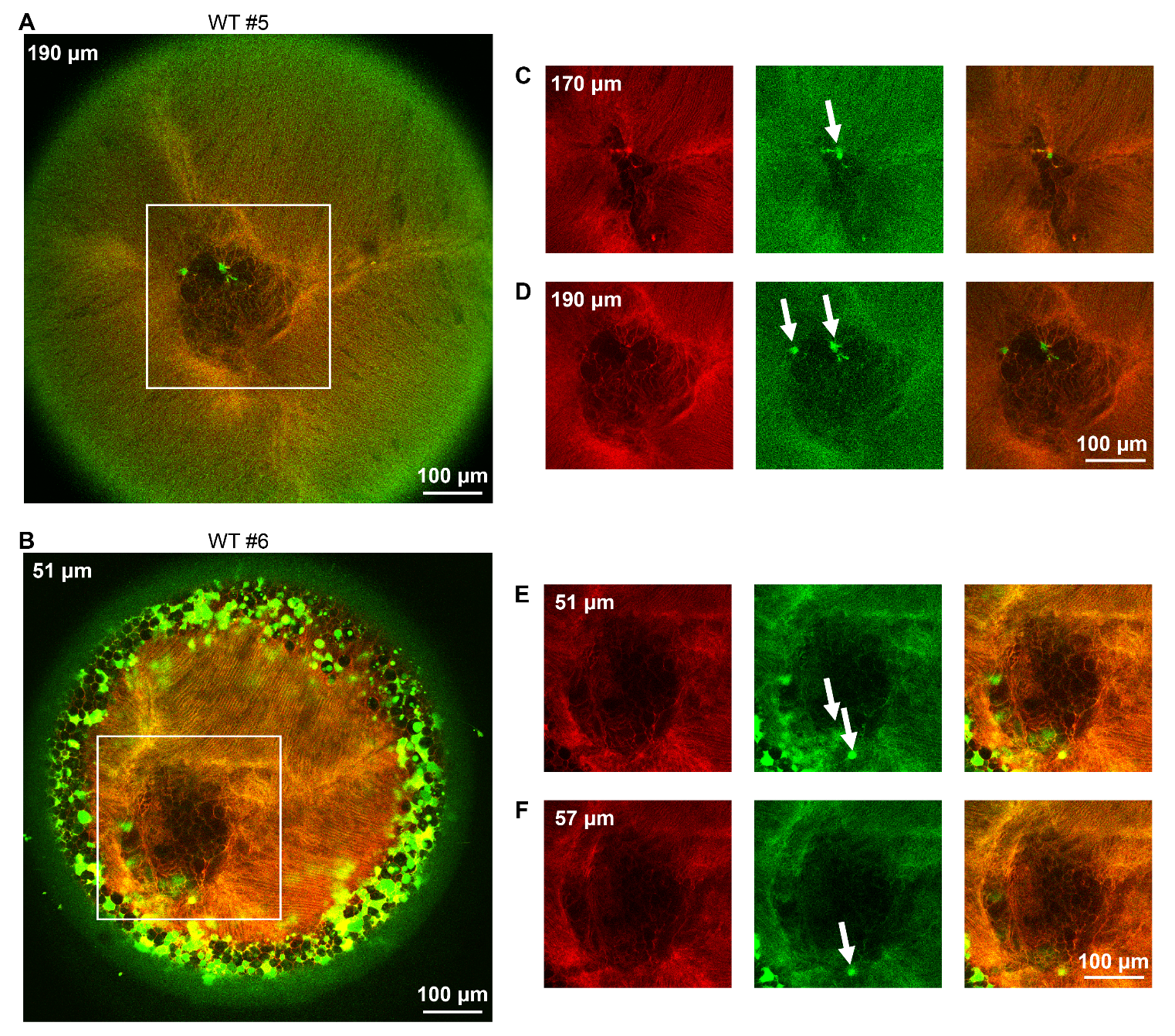

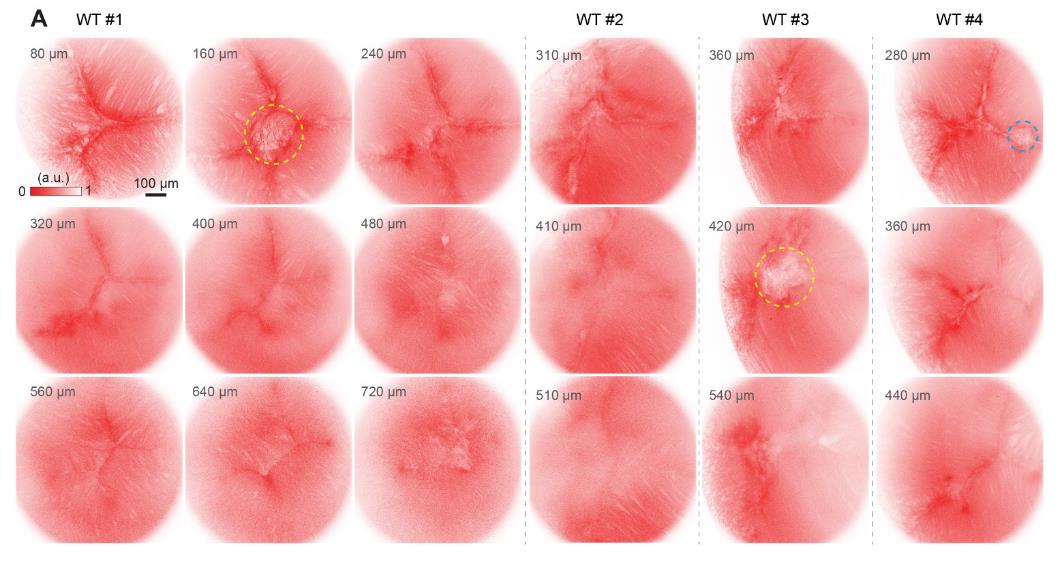


**Figure S6. 2PFM imaging of four wild-type (WT) lenses at different depths with void structure highlighted.** Yellow circles: central void at the conjunction of suture lines. Blue circle: lens void formed between two suture lines. Scalebar represents 100 µm and applies to all the images. Mice were 7–8 months old at the time of *in vivo* imaging; see Table S1 for details.

**Table S1. Animal information and experimental settings for all experiments.**

| *In vivo* imaging |  | Genotype | Sex | Age at imaging | Imaging parameters |
| --- | --- | --- | --- | --- | --- |
|  | WT #1 | tdTomato × WT | Male | 7 months and 11 days | FOV: 796.36 μm × 796.36 μm  Depth range: 600-980 μm  Z step size: 2 μm  Frames averaged at each depth: 15  Imaging wavelength: 1000 nm |
|  | WT #2 | tdTomato × WT | Male | 7 months and 11 days |  |
|  | WT #3 | tdTomato × WT | Male | 7 months and 11 days |  |
|  | WT #4 | tdTomato × WT | Male | 7 months and 11 days |  |
|  | KO #1 | L3: LCTL (-/-) × LCTL (-/-) | Female | 8 months and 2 days |  |
|  | KO #2 | L3: LCTL (-/-) × LCTL (-/-) | Female | 8 months and 2 days |  |
|  | KO #3 | L3: LCTL (-/-) × LCTL (-/-) | Female | 8 months and 2 days |  |
|  | KO #4 | L3: LCTL (-/-) × LCTL (-/-) | Male | 8 months and 3 days |  |
| *Ex vivo* imaging | KO #2 | L3: LCTL (-/-) × LCTL (-/-) | Female | 13 months and 2 days | FOV: 796.36 μm × 796.36 μm  Depth range: 420 μm  Z step size: 4 μm  Frames averaged at each depth: 20  Imaging wavelength: 920 nm  Dye used: 2,000,000 MW FITC-Dextran  Incubation time: 24 h |
|  | WT #5 | tdTomato × WT | Male | 7 months and 29 days | FOV: 796.36 μm × 796.36 μm  Depth range: 520 μm  Z step size: 5 μm  Frames averaged at each depth: 20  Imaging wavelength: 920 nm  Dye used: 10,000 MW FITC-Dextran  Incubation time: 20 h |
|  | WT #6 | tdTomato × WT | Female | 3 months and 1 day | FOV: 796.36 μm × 796.36 μm  Depth range: 261 μm  Z step size: 3 μm  Frames averaged at each depth: 20  Imaging wavelength: 920 nm  Dye used: 10,000 MW FITC-Dextran  Incubation time: 20 h |
